# Invasive Pulmonary Aspergillosis in Non-Neutropenic Patients: An Evolving Clinical Paradigm

**DOI:** 10.3390/diagnostics16010034

**Published:** 2025-12-22

**Authors:** Rocco Morena, Helen Linda Morrone, Francesca Serapide, Alessandro Russo

**Affiliations:** 1 Department of Medical and Surgical Sciences, “Magna Graecia” University of Catanzaro, 88100 Catanzaro, Italy; 2 Infectious and Tropical Diseases Unit, “Renato Dulbecco” Teaching Hospital of Catanzaro, 88100 Catanzaro, Italy

**Keywords:** invasive aspergillosis, neutropenic, invasive lung infections, IPA

## Abstract

Invasive pulmonary aspergillosis (IPA), traditionally associated with severe immunosuppression and neutropenia, is increasingly reported among non-neutropenic patients. This epidemiological shift highlights the need for a revised understanding of IPA’s pathogenesis, clinical presentation, and management strategies. The rising incidence in these populations likely reflects improved diagnostic capabilities and recognition of additional predisposing factors. Although profound immunosuppression remains a key risk, even moderate alterations in innate or adaptive immunity can promote *Aspergillus* spp. invasion. This review summarizes current knowledge and recent advances in the diagnosis and treatment of IPA. Specifically, treatment strategies must be tailored to comorbidities, infection severity, and drug tolerance. Early diagnosis and prompt antifungal therapy are crucial for improving outcomes. Voriconazole remains the first-line treatment, though therapeutic drug monitoring is essential to ensure efficacy and minimize toxicity. Isavuconazole represents an effective alternative, offering comparable efficacy, improved safety, predictable pharmacokinetics, and convenient once-daily dosing. Liposomal amphotericin B serves as a valuable option in severe or refractory cases due to its broad-spectrum activity and reduced nephrotoxicity. Supportive measures—such as respiratory optimization, comorbidity management, and immunomodulatory therapies—are integral to care. Prognosis depends on infection extent, immune status, and timeliness of therapy. Emerging antifungal agents, including olorofim, ibrexafungerp, and fosmanogepix, show promise against resistant *Aspergillus* species, expanding treatment options. Overall, IPA management in non-neutropenic patients requires a multidisciplinary, patient-centered approach integrating established antifungals, supportive care, and novel therapeutic advances.

## 1. Introduction

Invasive pulmonary aspergillosis (IPA), historically recognized as a hallmark opportunistic infection in severely immunocompromised individuals, particularly those with neutropenia, is increasingly documented in non-neutropenic patient populations [1,2]. This epidemiological shift necessitates a refined understanding of the pathogenesis, clinical manifestations, and diagnostic and therapeutic strategies pertinent to this evolving clinical paradigm. The heightened incidence of IPA in non-neutropenic patients may be attributed to several factors, including advancements in diagnostic modalities and an expanded recognition of predisposing conditions [3]. While severe immunosuppression remains a significant risk factor, perturbations in innate and adaptive immunity, even in the absence of absolute neutropenia, can facilitate *Aspergillus* spp. Invasion [4]. In this review we discussed all the aspects and state of the art of management of IPA.

## 2. Epidemiological and Pathophysiological Considerations

The paradigm of IPA has undergone a significant transformation, shifting from a disease predominantly associated with profound neutropenia to a condition increasingly recognized in non-neutropenic individuals [5,6,7,8]. This epidemiological shift calls for a comprehensive understanding of the underlying pathophysiological mechanisms that contribute to the susceptibility of these patients. Historically, IPA was considered a hallmark opportunistic infection in patients with severe immunosuppression, particularly those undergoing hematopoietic stem cell transplantation or intensive chemotherapy. However, more recent epidemiological data reveal a growing incidence of IPA in patients without absolute neutropenia [3,9,10]. This trend is attributed to several factors, including advancements in diagnostic techniques, increased awareness of the disease, and the expanding use of immunosuppressive therapies [11].

Risk stratification in non-neutropenic patients is crucial for early identification and intervention. Several predisposing factors have been identified, including chronic obstructive pulmonary disease (COPD), diabetes mellitus, long-term corticosteroid therapy, structural lung diseases, critical illness, and the use of immunomodulatory agents [12]. These factors contribute to a state of relative immunosuppression, which, while not as profound as in neutropenic patients, is sufficient to increase susceptibility to *Aspergillus* infection [10].

The pathogenesis of IPA in non-neutropenic patients is complex and involves a delicate interplay between host immunity and fungal virulence. While the absence of absolute neutropenia suggests a functional immune system, subtle perturbations in both innate and adaptive immunity can compromise host defenses [13,14]. Key risk factors include the following.

### 2.1. Compromised Pulmonary Integrity

Structural lung abnormalities represent a key risk factor for IPA in non-neutropenic patients, as they impair mucociliary clearance, reduce perfusion, and allow persistence and germination of inhaled conidia. These anatomical alterations disrupt epithelial integrity and local immune responses, facilitating progression from colonization to invasive disease [15].

Post-tuberculous cavities are strongly associated with IPA due to poor vascularization and retained necrotic material; characteristic CT features include thick-walled cavities and air-crescent sign. Bronchiectasis is similarly predisposed to *Aspergillus* colonization through mucus stasis and recurrent inflammation, often visualized radiologically as cystic or varicose dilatation with mucus impaction [16].

In COPD and emphysematous disease, chronic epithelial injury, bullae, and altered airway physiology create favorable conditions for fungal persistence, with CT imaging frequently showing cavitated nodules or consolidations. Pulmonary fibrosis, whether idiopathic or post-infectious, further increases risk by distorting lung architecture and impairing alveolar defense [15].

Finally, post-viral lung injury—particularly following influenza or SARS-CoV-2 infection—has increasingly been recognized as a relevant substrate for invasive pulmonary aspergillosis, largely due to epithelial disruption, impaired mucociliary clearance, and immune dysregulation. In affected patients, radiologic findings include ground-glass opacities or consolidations, while cavitary changes may occasionally develop in the context of fungal superinfection [17].

Overall, these forms of parenchymal damage establish an anatomical niche in which *Aspergillus* can overcome host defense mechanisms, even in the absence of classical immunosuppression.

### 2.2. Dysregulated Innate Immunity

The innate immune system plays a crucial role in the initial defense against *Aspergillus*. Outside of neutropenia, the dysregulation of other main actors and pathways of innate immune response can compromise the host defence against aspergillosis.

#### 2.2.1. Impaired Alveolar Macrophage Function

Alveolar macrophages are the first line of defense against inhaled *Aspergillus* spores. In patients with chronic lung diseases or those receiving corticosteroids, macrophage function impairment can lead to reduced phagocytosis and killing of fungal spores [18,19].

#### 2.2.2. C-Type Lectin Receptor (CLR) Alterations

Myeloid CLRs such as mannose receptor, Dectin-1 and Dectin-2 play a crucial role in antifungal immunity by recognizing components of the fungal cell walls (beta-glucan, mannans); the ligand–receptor binding induces cascade pathways (NFkB) involved in the activation of innate inflammatory response [20,21]. Dectin-1 deficient murine models infected by *Aspergillus* appeared to mount an excessive yet inefficient inflammatory response, with low IL-15 levels and consequent dampened NK activation [22].

#### 2.2.3. Defective Toll-like Receptor (TLR) Signaling

TLRs recognize fungal pathogen-associated molecular patterns (PAMPs) and initiate downstream signaling cascades that activate immune responses. Genetic or acquired defects in TLR signaling may compromise the ability to recognize and respond to *Aspergillus* [23].

#### 2.2.4. Neutrophil Dysfunction

In some conditions neutrophil dysfunction can occur while neutrophil count remains within range. Factors such as hyperglycemia in diabetic patients or chronic inflammation in COPD can impair neutrophil chemotaxis, phagocytosis, and oxidative burst activity [24].

#### 2.2.5. Th1/Th2 Imbalance

A shift towards a Th2-dominant immune response, as seen in allergic bronchopulmonary aspergillosis (ABPA), can be predisposed to invasive disease. Th1 responses are crucial for controlling fungal infections, while Th2 responses can promote inflammation and tissue damage [25,26].

#### 2.2.6. Immunomodulatory Therapies

The increasing use of immunomodulatory agents, such as tumor necrosis factor (TNF) inhibitors and other biologics, can disrupt normal immune homeostasis and increase susceptibility to IPA. These therapies can interfere with T cell function, cytokine production, and other immune mechanisms [27].

### 2.3. Critical Illness-Associated Immunosuppression

Patients in Intensive Care Units (ICUs) often experience a state of critical illness-associated immunosuppression, characterized by immune dysregulation and increased susceptibility to infections. The previous classification of systemic inflammatory response syndrome led to immune paralysis, characterized by impaired T cell function and increased susceptibility to opportunistic infections [28]. The progression to acute respiratory distress syndrome is associated with significant pulmonary inflammation and immune dysregulation, creating a favorable environment for *Aspergillus* invasion [29]. Finally, prolonged mechanical ventilation can disrupt normal pulmonary defenses and increase the risk of nosocomial infections, including IPA [30].

### 2.4. Host Genetic Factors

Genetic susceptibility plays an increasingly recognized role in determining which individuals tend to progress from *Aspergillus* colonization to invasive disease. Variants affecting genes involved in innate immune recognition, cytokine regulation, and antifungal effector pathways may significantly compromise host defense mechanisms, even when leukocyte counts are normal [31].

One of the most clinically relevant genetic defects associated with fungal vulnerability is CARD9 deficiency. CARD9 acts downstream of multiple CLRs, and is essential for activation of NFkB pathways, induction of IL-17 signaling, and recruitment of neutrophils to sites of fungal invasion [32]. Loss-of-function mutations in CARD9 impair innate immune activation, dampen cytokine/chemokine production, and reduce effective fungal clearance. Clinically, CARD9 deficiency has been strongly associated with severe and recurrent invasive fungal infections—including invasive aspergillosis—in otherwise immunocompetent individuals [33].

Beyond CARD9, additional genetic variants influence susceptibility. For example, polymorphisms in CLR genes (e.g., Dectin-1 Y238X) have been linked to impaired antifungal immunity [34]. Moreover, deficiency or low levels of serum opsonins such as Mannose-Binding Lectin (MBL), coded by the MBL2 gene, have been associated with increased risk of invasive aspergillosis in human cohorts [35].

In summary, these data highlight that genetic predisposition, particularly mutations or variants in CARD9, CLRs, or MBL, represents a critical yet often underestimated factor in the development of IPA in non-neutropenic individuals. Integrating genetic analysis into risk models and clinical protocols could improve patient stratification and guide personalized preventive and therapeutic strategies.

### 2.5. COVID and H1N1 Associated Invasive Pulmonary Aspergillosis

The dysregulated immune response and the epithelial damage observed in severe viral pneumonias are both predisposing factors to secondary bacterial and fungal infections. In particular, during the influenza and SARS-CoV-2 pandemics an increased incidence of IPA in patients with severe viral diseases posed a further challenge in the management of these patients and resulted in higher mortality rates leading to the definition of Influenza associated pulmonary aspergillosis (IAPA) and COVID-19 associated pulmonary aspergillosis (CAPA) [36,37].

The epidemiological shift towards increased IPA incidence in non-neutropenic patients underscores the complexity of host–pathogen interactions. A multifaceted approach, considering compromised pulmonary integrity, dysregulated innate and adaptive immunity, critical illness-associated immunosuppression, and host genetic factors, is essential for understanding the pathogenesis of IPA in this evolving clinical paradigm. Further research is needed to refine risk stratification strategies and develop targeted interventions to improve outcomes in these patients.

Table 1 summarizes the main pathophysiological mechanisms of IPA.

## 3. Clinical Presentation and Diagnosis

The clinical presentation of IPA in non-neutropenic patients can be heterogeneous, often mimicking other pulmonary pathologies and with frequent overlap of symptoms with other pulmonary pathologies. This diagnostic challenge necessitates a comprehensive approach, combining clinical evaluation with advanced imaging and microbiological techniques. In contrast to the fulminant presentation often observed in neutropenic patients, IPA in non-neutropenic individuals may present with a more insidious onset. Symptoms can range from non-specific respiratory complaints to more severe manifestations, depending on the extent of pulmonary involvement and the underlying comorbidities [10,47]. Respiratory symptoms can be very common with cough, which may be productive or non-productive, dyspnea, pleuritic chest pain, and hemoptysis that, although less frequent, is a significant indicator of angioinvasion and should raise suspicion for IPA [48]. Of importance, variability in clinical presentation is a milestone of IPA clinical picture. Patients with pre-existing lung diseases, such as COPD or bronchiectasis, may experience exacerbations of their underlying condition, making it difficult to distinguish IPA from other causes of respiratory deterioration [49]. As a matter of fact, the diagnosis of IPA in non-neutropenic patients requires a combination of clinical, radiological, and microbiological assessments.

In summary, diagnostic challenges arise from the non-specificity of symptoms and the limitations of traditional diagnostic methods and include the following.

### 3.1. Radiological Imaging

High-Resolution Computed Tomography (HRCT): HRCT is the cornerstone of radiological diagnosis [7,50]. It can reveal characteristic findings, including:

• Pulmonary nodules, which may be solitary or multiple.
• Halo sign, which represents a zone of ground-glass attenuation surrounding a nodule, indicative of hemorrhagic infarction [51].
• Air crescent sign, which signifies the separation of necrotic tissue from surrounding viable lung parenchyma.
• Cavitation, which may occur in areas of pulmonary necrosis.
• Infiltrates.

It is very important to know that in non-neutropenic patients, these classic signs might be less defined.

### 3.2. Microbiological Investigations

Culture of *Aspergillus* spp. from respiratory specimens, such as sputum or bronchoalveolar lavage (BAL) fluid, is essential for confirming the diagnosis. However, the sensitivity of culture may be limited, particularly in patients with non-productive cough [52].

The Galactomannan (GM) enzyme immunoassay detects a component of the *Aspergillus* cell wall in serum or BAL fluid. It is a valuable tool for early diagnosis, but its sensitivity and specificity can vary [53,54,55].

Beta-D-Glucan (BDG) assay detects a component of the fungal cell wall present in various fungal species, including *Aspergillus*. It is a useful screening test, but it is not specific for *Aspergillus* [56,57,58].

Polymerase Chain Reaction (PCR)-based assays can detect *Aspergillus* DNA in respiratory specimens or blood. PCR offers high sensitivity and specificity, but its availability may be limited [59].

### 3.3. Histopathological Examination

Lung biopsy remains the gold standard for definitive diagnosis. It allows for direct visualization of fungal hyphae and tissue invasion. However, it is an invasive procedure and may not be feasible in all patients [7].

Bronchoscopy with BAL, or when possible, with transbronchial biopsy, is less invasive, but can give valuable samples.

### 3.4. Clinical Laboratory Findings

While not specific, laboratory findings such as elevated inflammatory markers (e.g., C-reactive protein) and leukocytosis may support the diagnosis of IPA.

Then, the overlapping symptoms of IPA with other pulmonary infections and non-infectious conditions can make diagnosis challenging. The sensitivity of microbiological tests may be limited, particularly in patients with non-productive cough or those who have received prior antifungal therapy. The interpretation of radiological findings requires expertise, as other pulmonary pathologies may mimic IPA. Of importance, the clinical presentation of IPA in non-neutropenic patients is diverse and often non-specific. A comprehensive diagnostic approach, combining clinical evaluation, radiological imaging, and microbiological investigations, is essential for timely and accurate diagnosis.

Table 2 summarizes diagnostic modalities of IPA in Non-Neutropenic Patients.

## 4. Management of IPA: Therapeutic Strategies

The management of Invasive Pulmonary Aspergillosis (IPA) in non-neutropenic patients presents unique challenges, requiring a nuanced approach to therapeutic strategies and a careful consideration of prognostic implications. Unlike the standardized protocols often employed in neutropenic populations, treatment in non-neutropenic patients must be tailored to individual risk factors, comorbidities, and the severity of infection. The successful management of IPA hinges upon the prompt initiation of appropriate antifungal therapy, aiming to impede fungal proliferation and facilitate patient recovery. The therapeutic landscape for IPA encompasses a range of antifungal agents, each with distinct mechanisms of action and clinical applications [3,60,61].

The primary goal of therapy is to eradicate the fungal infection while minimizing treatment-related toxicity. This requires a combination of antifungal agents, supportive care, and, in some cases, surgical intervention [62].

Voriconazole is generally considered the first-line antifungal agent for IPA due to its broad-spectrum activity and favorable pharmacokinetic profile. It exhibits excellent tissue penetration and is available in both intravenous and oral formulations. Therapeutic drug monitoring (TDM) is crucial for optimizing triazole therapy, particularly voriconazole, due to its nonlinear pharmacokinetics and potential for drug interactions [63]. Monitoring trough levels helps ensure adequate drug exposure while minimizing the risk of toxicity [64].

Isavuconazole has emerged as a significant advancement in the therapeutic armamentarium against invasive pulmonary aspergillosis, offering a valuable alternative to established antifungal agents [65]. As a triazole antifungal, isavuconazole shares a similar mechanism of action with voriconazole, targeting the synthesis of ergosterol, a crucial component of the fungal cell membrane. This disruption leads to membrane instability and ultimately fungal cell death [66]. Clinical studies have demonstrated isavuconazole’s efficacy in the treatment of IPA, with results comparable to those achieved with voriconazole [67,68]. Notably, isavuconazole has shown promise in patients who may not tolerate or respond adequately to other antifungal therapies. Its favorable pharmacokinetic profile, characterized by predictable absorption and minimal variability, contributes to its ease of use and reduces the need for intensive therapeutic drug monitoring [69]. One of the key advantages of isavuconazole lies in its improved tolerability compared to some other triazole antifungals. Clinical studies have reported a lower incidence of adverse events, particularly visual disturbances and neurological side effects, which can be associated with voriconazole [67,68,69]. This enhanced tolerability can significantly improve patient compliance and quality of life during treatment [70,71].

Furthermore, isavuconazole exhibits a broader spectrum of activity against various *Aspergillus* species, including those with reduced susceptibility to other antifungals. This expanded coverage is particularly relevant in the context of emerging antifungal resistance, which poses a growing challenge in the management of invasive fungal infections [67]. Isavuconazole’s formulation as a water-soluble prodrug, isavuconazonium sulfate, allows for both intravenous and oral administration, providing flexibility in treatment strategies. The oral formulation offers a convenient option for outpatient therapy or step-down therapy following initial intravenous treatment, facilitating a seamless transition of care. In clinical practice, isavuconazole has proven to be a valuable option for both primary and salvage therapy of IPA. Its efficacy and tolerability profile make it a suitable choice for a wide range of patients, including those with comorbidities or those at risk of drug interactions [69]. However, careful consideration of potential drug interactions is still warranted, as isavuconazole can interact with certain medications metabolized by the cytochrome P450 enzyme system. Ongoing research continues to explore the optimal use of isavuconazole in IPA, including its role in combination therapy and its efficacy against specific *Aspergillus* species. As clinical experience with isavuconazole grows, its place in the therapeutic landscape of IPA is becoming increasingly well-defined, offering clinicians a valuable tool in the fight against this challenging infection [66,72,73,74,75].

Liposomal amphotericin B has secured a pivotal role in the management of invasive pulmonary aspergillosis, particularly in scenarios where other antifungal agents are contraindicated, ineffective, or poorly tolerated. As a lipid-based formulation of amphotericin B, this medication offers a critical advantage: it minimizes the severe toxicities associated with conventional amphotericin B deoxycholate, especially nephrotoxicity [76]. The mechanism of action of liposomal amphotericin B remains rooted in its ability to bind to ergosterol, a key component of the fungal cell membrane. However, the liposomal encapsulation significantly alters its pharmacokinetics and pharmacodynamics. The lipid carrier preferentially delivers the drug to fungal cells, reducing its exposure to host tissues, especially the kidneys. This targeted delivery substantially mitigates the risk of renal impairment, a major concern with conventional amphotericin B [77,78]. In the context of IPA, liposomal amphotericin B serves as a valuable first-line therapy in patients with severe disease, those with renal dysfunction, or those who have experienced adverse reactions to other antifungals. It is also frequently employed as salvage therapy when initial treatment with voriconazole or isavuconazole fails to achieve a satisfactory response. Clinical studies have demonstrated the efficacy of liposomal amphotericin B in achieving clinical and radiological improvement in patients with IPA. Its broad-spectrum activity against various *Aspergillus* species, including those with reduced susceptibility to triazoles, makes it a reliable option in challenging cases. The dosing of liposomal amphotericin B is typically higher than that of conventional amphotericin B, reflecting its improved tolerability. However, careful monitoring of renal function and electrolyte levels remains essential, particularly in patients with pre-existing kidney disease or those receiving concomitant nephrotoxic medications [79,80,81]. While liposomal amphotericin B offers a significant safety advantage over conventional amphotericin B, it is not without its own set of potential adverse effects. Infusion-related reactions, such as fever, chills, tachycardia and rigors, can occur, although they are generally less severe than those associated with conventional amphotericin B. Premedication with antipyretics and antihistamines can help minimize these reactions [82,83]. In summary, liposomal amphotericin B stands as a cornerstone in the treatment of IPA, providing a potent and relatively well-tolerated antifungal option. Its unique pharmacokinetic profile and broad-spectrum activity make it a valuable asset in the management of this life-threatening infection, particularly in high-risk patient populations [76,77]. Of interest, in severe or refractory cases, combination antifungal therapy may be considered. This approach aims to enhance antifungal activity and broaden the spectrum of coverage. Combinations of triazoles with echinocandins or amphotericin B have been used [84,85,86]. Moreover, surgical resection may be considered in localized IPA, particularly in patients with solitary pulmonary nodules or cavitary lesions. It can be a valuable adjunct to antifungal therapy, especially in cases of drug resistance or persistent infection. Surgical debridement may be necessary in cases of invasive fungal sinusitis or other extrapulmonary manifestations of IPA [63,87].

Supportive care is essential for managing complications of IPA and underlying comorbidities. This includes optimizing respiratory support, managing fluid and electrolyte imbalances, and providing nutritional support. Management of underlying conditions, such as diabetes mellitus or COPD, is crucial for improving outcomes. Immunomodulatory therapy reduction, when possible, is also important. Of interest, adjunctive therapies play a supportive role in the management of IPA. These may include immunomodulatory agents, such as interferon-gamma, to enhance host immune responses, and granulocyte colony-stimulating factor (G-CSF) to stimulate neutrophil production and function. Corticosteroids may be used in carefully selected cases to manage inflammatory complications, but their use must be balanced against the potential risks of immunosuppression. The use of adjunctive therapies must be considered on a case-by-case basis. Close monitoring of patients with IPA is essential, including regular assessment of clinical signs and symptoms, imaging studies to evaluate the extent of pulmonary involvement, and laboratory tests to monitor antifungal drug levels and assess organ function. Supportive care measures, such as respiratory support and nutritional support, are also crucial for patient management [47,88,89].

The prognosis of IPA in non-neutropenic patients is influenced by several factors, including the severity of infection, underlying comorbidities, and the timeliness of diagnosis and treatment. Patients with extensive pulmonary involvement, angioinvasion, or extrapulmonary dissemination have a poorer prognosis. The presence of respiratory failure or sepsis significantly increases mortality risk. Patients with multiple comorbidities, such as diabetes mellitus, COPD, or chronic kidney disease, are at increased risk of complications and mortality. Immunosuppressive therapies, such as long-term corticosteroid therapy or immunomodulatory agents, can impair immune responses and worsen outcomes.

Early diagnosis and prompt initiation of appropriate antifungal therapy are crucial for improving outcomes [90,91,92]. Delays in diagnosis or treatment can lead to disease progression and increased mortality. The ability of the host immune system to mount an effective response against *Aspergillus* is a critical determinant of prognosis. Patients with impaired immune function, even in the absence of absolute neutropenia, are at increased risk of treatment failure and relapse. Of importance, the emergence of antifungal resistance can complicate treatment and worsen prognosis. Monitoring for drug resistance and adjusting therapy accordingly is essential [5,8,10].

Further research is needed to optimize therapeutic strategies and improve outcomes in non-neutropenic patients with IPA.

This includes:

• Developing more sensitive and specific diagnostic tools for early detection.
• Evaluating the efficacy of novel antifungal agents and combination therapies.
• Identifying biomarkers for predicting treatment response and prognosis.
• Developing personalized treatment approaches based on individual risk factors and comorbidities.

Then, the management of IPA in non-neutropenic patients requires a multidisciplinary approach, combining antifungal therapy, surgical intervention, and supportive care. Careful consideration of prognostic factors is essential for optimizing treatment strategies and improving patient outcomes [3,5,60,61,62,64].

## 5. Mechanisms of Azole Resistance in *Aspergillus* spp.

Azole antifungals, including itraconazole, voriconazole, and posaconazole, are the mainstay of treatment for *Aspergillus* infections. However, the emergence of azole resistance in *Aspergillus* species, particularly *Aspergillus fumigatus*, poses a significant clinical challenge [93]. Azole-resistant aspergillosis is associated with increased treatment failure, higher mortality rates, and limited therapeutic options [46,94].

Azole resistance in *Aspergillus fumigatus* is predominantly driven by point mutations and tandem repeats in the cyp51A gene, encoding the 14α-demethylase enzyme—the molecular target of triazole antifungal; these mutations are mostly developed due to widespread use of azole compounds as agricultural fungicides [95]. The main resistance mechanisms include:

1. **Point Mutations in cyp51A.**
  • Mutations such as G54, M220, and G448 alter azole binding, reducing drug efficacy.
2. **TR34/L98H and TR46/Y121F/T289A Mutations [96].**
  • The tandem repeat (TR) mutations are associated with environmental resistance due to agricultural azole use.
3. **Efflux Pump Overexpression.**
  • Increased expression of efflux pumps, such as ATP-binding cassette (ABC) transporters, reduces intracellular azole concentration.
4. **Biofilm Formation and Stress Responses.**
  • Biofilm production enhances fungal persistence and reduces azole penetration.

Azole resistance is increasingly reported worldwide, with notable prevalence in Europe, Asia, and the Americas. The resistance emerges in both clinical and environmental settings due to:

• Long-term azole therapy in patients with chronic aspergillosis.
• Antifungal prophylaxis with azole in hematologic patients.
• Environmental selection pressure from azole fungicides used in agriculture [46,93,96].

However, the increasing prevalence of azole-resistant *Aspergillus* strains has led to several critical repercussions. Azole resistance directly compromises the effectiveness of standard antifungal therapies, leading to higher mortality rates, particularly among immunocompromised individuals [46]. Patients with conditions such as organ transplants, hematological malignancies, or chronic lung diseases are especially vulnerable. The presence of azole resistance complicates treatment decisions. Clinicians are often forced to resort to alternative antifungal agents, which may exhibit greater toxicity, reduced efficacy, or higher costs. This necessitates more complex treatment regimens and closer patient monitoring. Identifying azole-resistant strains requires specialized laboratory testing, which can lead to delays in initiating appropriate therapy. These delays can have severe consequences, as aspergillosis can progress rapidly in susceptible individuals [46]. The environmental dissemination of azole-resistant *Aspergillus* spores is a growing concern. The use of azole fungicides in agriculture has been implicated as a significant driver of resistance development, creating a link between agricultural practices and human health [97]. This highlights the importance of a “One Health” approach to address antifungal resistance. The management of azole-resistant aspergillosis places a substantial economic burden on healthcare systems. The need for more expensive alternative therapies, prolonged hospital stays, and increased diagnostic testing contributes to rising healthcare costs. Of importance is the rise in azole resistance is limiting the amount of treatment options that clinicians have. This is a very concerning issue, because it makes it much harder to treat patients with aspergillosis.

In essence, azole resistance in *Aspergillus* has far-reaching consequences, affecting patient outcomes, clinical practice, and public health. Continuous surveillance, judicious antifungal use, and the development of novel therapeutic strategies are crucial to mitigating the impact of this growing threat.

## 6. Comparative Efficacy and Safety of Isavuconazole Versus Voriconazole

Although both isavuconazole and voriconazole are approved for the treatment of IPA, important differences in their pharmacological and safety profiles influence clinical decision-making. Isavuconazole is characterized by a long elimination half-life of approximately 100 to 130 h, which allows for once-daily maintenance dosing after an initial loading phase. In contrast, voriconazole requires twice-daily administration and displays nonlinear pharmacokinetics with high interindividual variability, often necessitating therapeutic drug monitoring to optimize exposure and minimize toxicity [97,98,99,100]. The simplified dosing schedule and lower potential for pharmacokinetic fluctuation make isavuconazole particularly advantageous for long-term treatment, especially in outpatient settings or in patients with limited access to therapeutic drug monitoring. From a safety perspective, isavuconazole has demonstrated a more favorable profile, with significantly lower rates of hepatotoxicity, visual disturbances, and dermatologic adverse events compared to voriconazole [101,102]. Additionally, unlike other triazoles, isavuconazole shortens rather than prolongs the QT interval, making it a safer option in patients with baseline QT prolongation or those receiving other QT-prolonging agents [103,104].

Importantly, these attributes have not compromised its efficacy. The phase III SECURE trial established non-inferiority of isavuconazole compared to voriconazole in terms of all-cause mortality and overall treatment response in patients with provenor probable IPA [104,105]. Real-world experience further supports its use, particularly in patients with comorbidities such as chronic liver disease or cardiac arrhythmias, where tolerability concerns may limit voriconazole use [106]. Taken together, these features position isavuconazole as a clinically valuable alternative for first-line or step-down therapy, offering practical advantages that may improve adherence, reduce monitoring burden, and minimize treatment-related toxicity.

## 7. Emerging Antifungal Agents: A New Era

In this scenario, the development of new antifungal agents with novel mechanisms of action and improved pharmacokinetic properties offers hope for overcoming these challenges.

Olorofim is an orotomide antifungal, which has a novel mechanism of action, targeting dihydroorotate dehydrogenase, an enzyme essential for pyrimidine biosynthesis. Olorofim has demonstrated potent in vitro and in vivo activity against *Aspergillus* species, including azole-resistant strains. It also has shown promise against other molds, such as *Scedosporium* and *Lomentospora* spp. This broadens the treatment options, particularly for patients with difficult-to-treat molds [107,108].

Ibrexafungerp is a triterpenoid antifungal that inhibits glucan synthase, an enzyme crucial for fungal cell wall synthesis. It exhibits activity against a wide range of fungi, including *Aspergillus* spp. Notably, it is available in both intravenous and oral formulations, providing flexibility in treatment administration. This is very helpful for step down therapy [109,110].

Fosmanogepix is a Gwt1 inhibitor. It has a novel mechanism of action with activity against molds that are also difficult to treat, such as *Fusarium* and *Scedosporium* spp. [111,112].

The emergence of these novel antifungal agents has significant clinical implications for the treatment of IPA. These new drugs offer the potential for improved outcomes in patients with IPA, particularly those with drug-resistant infections or comorbidities that limit the use of current therapies. The availability of antifungal agents with different mechanisms of action expands treatment options and allows for personalized therapy based on individual patient characteristics. Some of the newer agents exhibit improved safety profiles compared to older antifungals, potentially reducing treatment-related toxicity [107,108,109,111].

All these new antifungal agents, along with the current therapeutic options, are compared in Table 3.

In conclusion, the development of novel antifungal agents represents a promising advancement in the treatment of IPA. These agents offer the potential to overcome the limitations of current therapies and improve outcomes for patients with this life-threatening infection.

## Figures and Tables

**Table 1:** Pathophysiological mechanisms of invasive pulmonary aspergillosis.

Pathophysiological Aspect	Description
Host Immune Status	Non-neutropenic patients often have intact neutrophils but may have other immune dysfunctions, such as impaired macrophage or T-cell function (e.g., due to long-term corticosteroid therapy or chronic lung disease) [3].
Epithelial Barrier Disruption	Structural lung damage from conditions like COPD, influenza, or mechanical ventilation can facilitate fungal invasion [38,39].
Inflammatory Response	Unlike neutropenic patients, non-neutropenic individuals may exhibit excessive inflammation due to dysregulated immune activation, leading to tissue damage and worsened outcomes [40].
Role of Alveolar Macrophages	Macrophages play a critical role in *Aspergillus* clearance. Dysfunction due to long-term corticosteroid therapy or chronic lung disease can impair fungal killing [41].
Angioinvasion vs. Airway Invasion	In non-neutropenic patients, airway-centered invasion (bronchopulmonary aspergillosis) is more common, whereas angioinvasion is more frequent in neutropenic individuals [42].
Corticosteroid Impact	Corticosteroids suppress macrophage and dendritic cell function, leading to impaired fungal clearance and increased risk of invasive disease [43].
Pulmonary Comorbidities	Underlying lung diseases (e.g., COPD, asthma, bronchiectasis) create an environment favoring *Aspergillus* colonization and invasion [12].
Influenza/Viral Co-Infections	Viral infections (such as influenza or COVID-19) cause alveolar damage and immune dysregulation, increasing susceptibility to IPA [2,37,44].
Immunomodulatory Therapy	Patients receiving TNF inhibitors, anti-IL-6, or other immunosuppressants are at increased risk due to altered immune responses [45].
Delayed Diagnosis	Due to the lack of classical angioinvasive features, diagnosing IPA in non-neutropenic patients is often challenging, leading to delayed treatment and higher mortality [10,46].

**Table 2:** Diagnostic Modalities of IPA in Non-Neutropenic Patients.

Diagnostic Method	Sensitivity	Specificity	Advantages	Limitations
HRCT Scan [7]	Moderate	Moderate	Non-invasive, rapid	Nonspecific findings in non-neutropenic patients
BAL Culture [7]	Low to Moderate	High	Confirms presence of *Aspergillus*	Differentiating colonization from infection is difficult
Galactomannan (BAL) [53,54,55]	High	Moderate	High diagnostic value in BAL samples	False positives due to diet, antibiotics
(1→3)-β-D-Glucan [56,57,58]	High	Low to Moderate	Broad fungal detection	Cross-reactivity with other fungi, bacterial infections
*Aspergillus* PCR [59]	High	High	Rapid, sensitive detection	Lack of standardization across labs
Histopathology [7]	High	High	Gold standard	Invasive procedure

**Table 3:** Comparison of Current vs. Emerging Antifungal Treatments for IPA.

Drug Class	Agent	Mechanism of Action	Antifungal Spectrum	Key Benefits
**Current Treatments**				
**Triazoles**	Voriconazole	Inhibits ergosterol synthesis (CYP51A1 inhibition)	***Aspergillus*** **spp.**, ***Candida*** **spp.**	First-line therapy for IPA, oral & IV forms available
	Isavuconazole	Inhibits ergosterol synthesis	***Aspergillus*** **spp.**, ***Mucorales, Candida*** **spp.**	Broad spectrum, fewer side effects than voriconazole
**Polyenes**	Liposomal Amphotericin B	Binds to ergosterol, disrupting fungal cell membrane	***Aspergillus*** **spp.**, ***Mucorales, Candida*** **spp.**	Used in severe cases, broad spectrum, IV only
**Echinocandins**	Caspofungin, Micafungin, Anidulafungin	Inhibits β-glucan synthase (cell wall synthesis)	***Candida*** **spp.**, **limited** ***Aspergillus*** **spp. activity**	Salvage therapy, low toxicity, IV only
**Emerging Treatments**				
**Orotomides**	Olorofim	Inhibits pyrimidine biosynthesis (DHODH inhibition)	***Aspergillus*** **spp.**, ***Scedosporium*** **spp.**, ***Lomentospora*** **spp.**	Active against azole-resistant *Aspergillus*, oral bioavailability
**Triterpenoids**	Ibrexafungerp	Inhibits β-glucan synthase (cell wall synthesis)	***Aspergillus*** **spp.**, ***Candida*** **spp.**	Oral echinocandin, step-down therapy option
**Gwt1 Inhibitors**	Fosmanogepix	Inhibits Gwt1 enzyme, affecting GPI-anchored proteins	***Aspergillus*** **spp.**, ***Fusarium*** **spp.**, ***Scedosporium*** **spp.**	Active against difficult-to-treat molds, broad-spectrum potential
**Long-Acting Echinocandins**	Rezafungin	Inhibits β-glucan synthase	***Candida *** **spp.**, ***Pneumocystis*** **spp.**	Once-weekly dosing, improved pharmacokinetics
**Next-Gen Azoles**	New azole compounds	Improved CYP51A1 inhibition, fewer drug interactions	***Aspergillus *** **spp.**, ***Candida*** **spp.**	Better tolerability, possible inhaled formulations

## Data Availability

No new data were created or analyzed in this study. Data sharing is not applicable to this article.
